# Glycopolymeric Materials for Advanced Applications

**DOI:** 10.3390/ma8052276

**Published:** 2015-04-29

**Authors:** Alexandra Muñoz-Bonilla, Marta Fernández-García

**Affiliations:** 1 Departamento de Química Física Aplicada, Facultad de Ciencias, Universidad Autónoma de Madrid (UAM), C/ Francisco Tomás y Valiente 7, Cantoblanco, 28049 Madrid, Spain; 2 Instituto de Ciencia y Tecnología de Polímeros (ICTP-CSIC), C/ Juan de la Cierva 3, 28006 Madrid, Spain

**Keywords:** glycopolymers, biorecognition, anti-adhesion therapy, bioactive delivery, vaccines, sensors, imaging, diagnostics

## Abstract

In recent years, glycopolymers have particularly revolutionized the world of macromolecular chemistry and materials in general. Nevertheless, it has been in this century when scientists realize that these materials present great versatility in biosensing, biorecognition, and biomedicine among other areas. This article highlights most relevant glycopolymeric materials, considering that they are only a small example of the research done in this emerging field. The examples described here are selected on the base of novelty, innovation and implementation of glycopolymeric materials. In addition, the future perspectives of this topic will be commented on.

## 1. Introduction

It is well known that carbohydrates located in the cell membrane as glycoproteins, glycolipids or glycans play a crucial role in many physiological as well as pathological events, including cellular proliferation and cancer metastasis, intercellular communication, recognition processes, adhesion, *etc.* This is mainly as a result of specific interactions of the carbohydrates with protein receptors such as enzymes or lectins that triggering the above mentioned biological functions. This molecular recognition ability is greatly enhanced by multivalency or the so-called glycocluster effect [1,2], commonly found in natural glycoproteins or glycolipids clusters that strongly recognize sugar-binding proteins. 

Numerous investigations have been focused on the preparation of synthetic glycopolymers with pendant sugar moieties along the polymer backbone or at the end of the chain, which are able to interact with lectins through multivalent interactions, mimicking natural biomolecules. Especially interesting are the controlled polymerization techniques and efficient chemical reactions such as click chemistry to tune the architecture of the glycopolymers and optimize the recognition process [3,4,5]. The design of the glycopolymer is essential, because the strength of the binding strongly depends on the type of sugars, anomeric status, and linkage position and the linker that connects the carbohydrate to the polymer backbone, and also on the density of sugars, degree of polymerization and branching [6]. For example, by increasing the polymer length, then the valence, polymers can access to multiple binding sites in proteins, thereby increasing their affinity. Nevertheless, when all accessible binding sites are occupied, further increase in polymer length will not yield enhancements in their interaction. Moreover, more flexible backbones and linkers allow polymer to adopt a conformation/orientation that leads to effective interactions. It is important to mention that the interaction between glycopolymer and lectin is exclusive for each binomial team; that is, the best polymer structure for a particular lectin is not necessarily the optimum structure for other lectins, even those with the same carbohydrate specificity.

Synthetic polymeric techniques such as reversible addition-fragmentation chain transfer polymerization (RAFT) [7] and atom transfer radical polymerization (ATRP) [8] allow the development of well-defined glycopolymers of a variety of composition and topologies, including homopolymers, statistical and block copolymers. 

In the last years, efforts were mainly dedicated to the synthesis of new glycopolymer structures and their use as biomimetic model to fundamentally investigate the specific carbohydrate-protein interactions. Nowadays, besides the synthetic development, glycopolymeric nanostructures, both polymer glyconanoparticles and hybrid nanoparticles are receiving more and more attention. Nanoparticles due to their size and high surface area have shown great potential in different fields, especially in biomedicine. Recently, research has demonstrated that the size and shape of the nanoparticles significantly influence their interactions with cells. A variety of approaches, such as grafting from or grafting on, have been used to prepare glyconanoparticles with different compositions, sizes and shapes to study and understand the interactions between particles and proteins and optimize their applicability [9]. 

Based on the extensive investigations of the biological activity of the glycopolymers and how their structure affects the binding with lectins the focus of research has moved more to the pure applications of glycopolymers, which also contribute to amplify the knowledge in the recognition process. Lately, advancements in the applications of glycopolymers have shown an explosion, with the consolidation and advance of several uses, such as target delivery systems and also emerging new applications. This article will examine the most common and recent applications of glycopolymers particularly focused in biomedical and biological uses. 

## 2. Biomimetic Model to Investigate Carbohydrate-Protein Interactions

As mentioned above, glycopolymers can strongly interact with specific proteins, lectins, by the multivalent glycocluster effect. There are many fundamental examples that investigate glycopolymer-lectin recognition [10,11,12,13,14,15,16,17,18,19,20,21,22,23], especially with Concanavalin A, Con A, and *Ricinus Communis* Agglutinin 120, RCA_120_, mainly by UV-Vis or fluorescence spectroscopies; in the latter when either the lectin or the glycopolymer are fluorescence-labeled. It is well-known that Con A, which is a tetramer with four carbohydrate binding sites, specifically binds to glycopolymers containing mannosyl and glucosyl residues while RCA_120_ lectin interacts to those containing galactosyl residues. These residues have to be present in a specific form, otherwise there will be no interactions. This is the case of gluconolactone derivatives, where the glucose moieties are in an open-form and the interactions are not observed either with RCA_120_ or with Con A [24,25].

Moreover, the binding abilities of glycopolymers have been also measured by microarray and surface plasmon resonance (SPR) techniques [26,27,28,29,30,31]. Microarray technique of biomolecules, also called biochip, is a collection of microscopic spots of these molecules attached to a solid surface [32,33]. Meanwhile, surface plasmon resonance sensing is also a powerful and quantitative probe of the interactions of a variety of biopolymers with various ligands. It provides a means not only for identifying these interactions and quantifying their equilibrium constants, kinetic constants and underlying energetics, but also for employing them in very sensitive biochemical assays [34,35].

**Figure 1:**
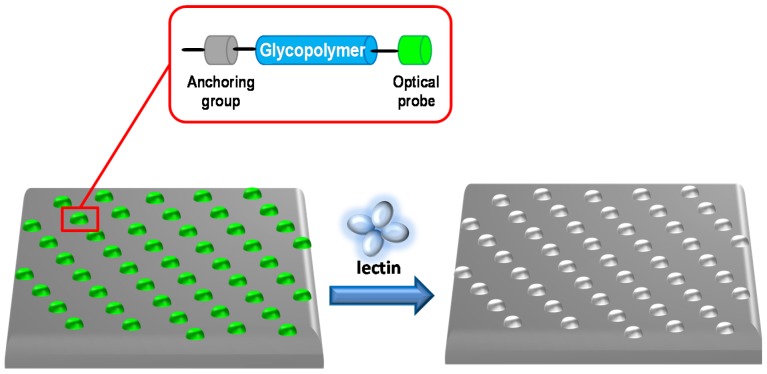
Schematic representation of glycopolymers anchored on chips and their interaction.

In this context, the glycopolymers can be efficiently attached to surfaces, where they approximate physiological cell–cell and cell–extracellular matrix interactions and retain the ability to engage proteins. This is the case of chondroitin sulfate glycomimetic polymers that present sulfated disaccharide units, which mimic the multivalent architecture of native glycosaminoglycans chains [27]. Glycopolymers were attached to microarray surfaces with a high-precision contact-printing robot delivering nanoliter volumes of the biotin-labeled glycopolymers to streptavidin-coated slides, yielding spots of approximately 200 mm in diameter (see Figure 1). 

Bertozzi group has used the microcontact printing (mCP) technique to covalently array the glycopolymers on azide-functionalized biochips in well-defined patterns using a poly(dimethylsiloxane) stamp with approximately 2 mm circular structures [31]. They tested the binding effectiveness of *Helix Pomatia* Agglutinin (HPA) lectin, proving the specific interaction. 

**Figure 2:**
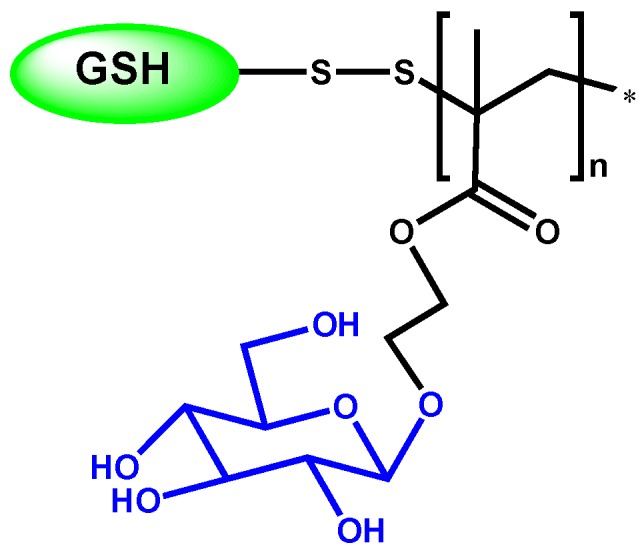
Tripeptide reduced glutathione-bioconjugate glycopolymer structure.

Narain group has studied the recognition ability of glyconanoparticles with immobilize lectins, *i.e.*, *N*-acetyl β-D-glucosaminoside decorated gold nanoparticles, which specifically interact with wheat germ agglutinin (WGA) lectin, whereas α-D-mannoside derivatives specifically interact with Con A lectin. The glycopolymer structure gives the capability of lectin interaction, but can also infer other properties. This is the case of glycopolymers obtained by RAFT process, where the polymer is conjugated with tripeptide reduced glutathione (GSH) by thiol-disulfide exchange reaction [36]. The resulting disulfide-linked GSH bioconjugate polymer (Figure 2) possesses antioxidant character, with prolonged radical scavenging activity. This is because of GSH, which can protect cells from oxidative stress through scavenging singlet oxygen, hydroxyl radicals, and superoxide radicals. Moreover, the specific recognition with Con A lectin is enhanced about three times when it is conjugated with a GSH, other than on its own.

**Figure 3:**
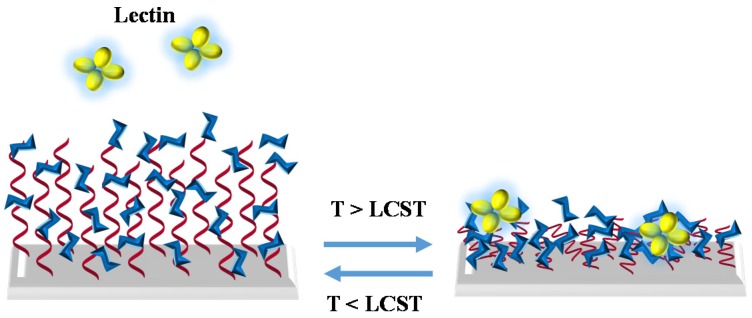
Representation of interaction between random thermoreversible glycopolymers attached to surface and lectin.

**Figure 4:**
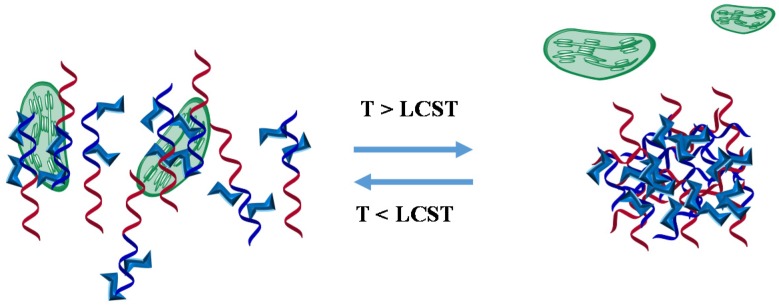
Representation of interaction between block thermoreversible glycopolymers and bacteria in water solution.

Stimuli-responsive structures enlarge the properties of glycopolymers and make the binding properties more versatile. For example, thermoreversible glycopolymers used to control lectin interaction or bacterial aggregation [37,38], where the binding between glycopolymer and lectin or bacteria is regulated by the polymer thermoreversibility. In the first case, the random glycopolymer based on *N*-isopropyl acrylamide (NIPAAm) and *N*-acryloyl glucosamine (AGA) is grafted from the honeycomb surface. Below the lower critical solution temperature (LCST) of the surface, the conjugation is switched off, while above the LCST the surface grafted glucose moieties bind strongly to Con A (Figure 3). Contrary effect is observed in the second example in which the block glycopolymer based also on NIPAAm is in solution. In this situation, when temperature is below its LCST, the chains are extended and display glucose moieties in solutions, then it is able to bind to the cell of *Escherichia coli* bacteria, whereas above LCST, the glycopolymer is presented in a globular state, which hides the carbohydrate moieties (Figure 4).

## 3. Anti-Adhesion Therapy

Currently, one of the most attracting and emerging applications of glycopolymers is anti-adhesion therapy in which glycopolymers behave as antagonists to interfere with the binding of lectins from a pathogen to the host organism and, therefore, prevent infection from occurring. Glycopolymers have been designed to act against infections by different AB_5_ toxins, including the cholera toxin (CT), Shiga toxins, and the heat-labile enterotoxin (LT). Different degrees of carbohydrate incorporation have been obtained by post-modification of poly(L-glutamic acid) (PGA) backbone with *N*-(ԑ-aminocaproyl)-β-D-galactosylamine over the β-D-galactosylamine. The results indicate that higher accessibility of galactose in *N*-(ԑ-aminocaproyl)-β-D-galactosylamine improves the inhibition of binding [39,40]. 

Moreover, glycopolymers bearing globotriose or lactose and acrylamide have been used to evaluate the inhibitory effects on cytotoxicity of Shiga toxins (Stxs; Stx1 and Stx2). The results showed that glycopolymers having globotriose units exhibited inhibitory effect on cytotoxicity of Stx1, while glycopolymers having small amount of acrylamide exhibited inhibitory effect on cytotoxicity of Stx2. Also, the glycopolymer, having lactose, was able to bind Stx1, showing inhibitory effect on its cytotoxicity [41]. The potential of glycopolymer containing mannose to interact with human DC-SIGN, thereby inhibiting the interactions between DC-SIGN and the HIV envelope glycoprotein gp120 was also reported [42]. They prepared a library of glycopolymers and demonstrated that simple structures effectively prevent infections. This study has a considerably importance because the DC-SIGN is also implicated in the action mechanism of other viruses including Ebola and hepatitis C. 

Following the same strategy, different glycopolymers were synthetized by post-modification to inhibit bacterial toxins such as cholera [43,44,45]. They proved the importance of the glycopolymer structure in terms of linker length and branching, carbohydrate density and molecular weight on the inhibition of the bacterial toxins. Specifically, longer linkers increase inhibition of the B subunit of cholera toxin, which is attributed to the depth of the binding pocket. Moreover, Gibson group also demonstrated that branched side chains into the linker in galacto-terminal polymers increase binding affinity to their corresponding lectins, relative to simple monosaccharides.

## 4. Bioactive Delivery Systems

**Figure 5:**
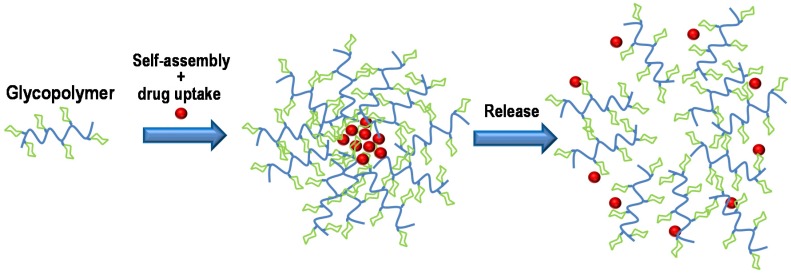
Scheme of glycopolymer self-assembly along with bioactive drug uptake and posterior release of the compound.

Drug delivery systems are one of the main applications of glycopolymers, since, theoretically, they do not produce undesirable side effects but can easily reach their targets and increase the solubility of the system. Most of the glycopolymer systems used in drug delivery are nanostructures, in particular micellar systems adequate for oral and intravenous delivery route of poorly water-soluble drugs (Figure 5).

**Figure 6:**
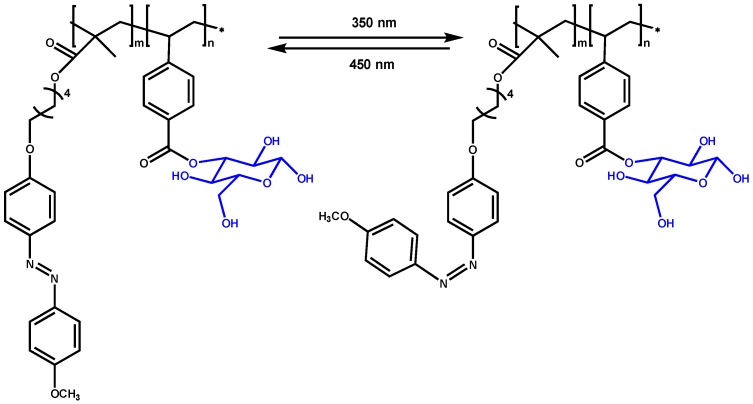
Light-induced isomerization of block copolymers poly(azobenzene methacrylate)-*b*-poly(3-O-4-vinylbenzoyl-D-glucopyranose), under alternate ultraviolet and visible irradiation for encapsulate-release process.

Especially interesting are stimuli responsive systems that allow smart strategies of delivery. For instance block glycocopolymer based on poly(azobenzene methacrylate) (PMAzo) and poly(3-O-4-vinylbenzoyl-D-glucopyranose) (PBG) was reported (Figure 6). Although this glycopolymer has not been used for bioactive delivery, they are able to encapsulate, release, and re-encapsulate water-insoluble Nile Red as model compound [46]. PMAzo-*b*-PBG glycopolymer self-assembles into spherical aggregates in water but UV irradiation destabilizes the aggregates as a result of the *trans*–*cis* isomerization of the MAzo units. The dissociated aggregates reunite to form tubular aggregates when the solution is irradiated at 450 nm.

Other glycopolymers have been used to form micelles through their self-aggregation to encapsulate gold nanoparticles [47]. In detail, maltoheptaose-*b*-polystyrene (MH-*b*-PS) can incorporate PS surface-functionalized gold nanoparticles. In this article, the influence of the preparation method of micelles on the particle size is described. Nanoprecipitation of the block copolymer solution, where the block copolymer molecules exist as well-swollen single chains into a large amount of selective solvent for carbohydrate segment, produces micelle nanoparticles of ~30 nm while the reverse procedure produces random aggregations and formed large micelles of ~80 nm.

Suriano *et al.* [48] have synthetized amphiphilic block glycopolymers containing D-glucose, D-galactose and D-mannose by metal-free organocatalyzed ring-opening polymerization. These glycopolymers were able to form micelles with particle sizes of 100 nm and low polydispersities, which are not toxic to asialoglycoprotein receptors (ASGP-R), positive HepG2 (human liver hepatocellular carcinoma) cells or ASGP-R negative HEK293 cells. These authors demonstrated the selectivity of galactose-containing micelles that deliver doxorubicin (DOX) more efficiently into HepG2 cells than in HEK293 cells. Moreover, the cytotoxicity of DOX against HepG2 cells was significantly increased when delivered using the galactose-containing micelles as compared to the free DOX formulation and the glucose-containing micelles. It is important to mention that the regioisomerism is crucial for binding properties. Glycopolymers carrying different constitutional isomers of the pendent sugar species, *i.e.* the glycosidic linkage of the galactose unit from the anomeric position, position 1, and position 6 shows different interaction. The first has strong binding to lectins of peanut (*Arachis hypogea*) agglutinin (PNA) and *Erythrina cristagalli* agglutinin (ECA), while the later does not. More significantly, they show binding behavior similar to the ASGP-R, but different internalization pathways in the HepG2 cell after ASGP-R mediated endocytosis [49].

**Figure 7:**
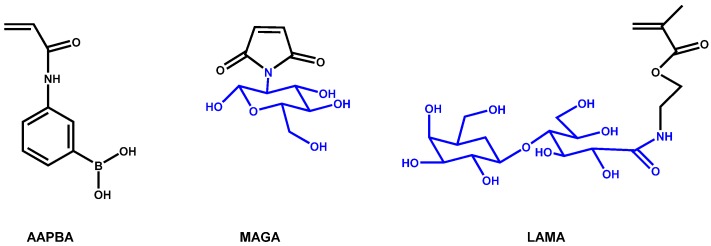
Different monomers used for copolymerization.

Glycopolymers can also be used as nasal drug delivery systems for delivery of proteins and peptides, since they can avoid degradation in gastrointestinal tract and metabolism by liver enzymes and are non-toxic. However, nasal delivery has to overcome the self-defense mechanisms, such as mucocillary clearance, ciliary beat frequency and inflammation, and enzymatic barrier, which affect the permeability of drugs through the nasal mucosa. The boronic acid and its derivatives are known to possess the ability to reversibly interact with diols, sugars, and glycoproteins, and transport saccharide across lipid bilayers. However, the main drawback of these materials is the cytotoxicity. In this sense, glycopolymers have been proposed to increase the biocompatibility and hydrophilicity of these compounds. Jin *et al.* [50] described the synthesis of amphiphilic copolymers based on 3-acrylamidophenylboronic acid (AAPBA) with maleimide-glucosamine (MAGA) at different ratios (Figure 7). 

They demonstrated the biocompatibility and, therefore, the copolymers are innocuous and do not have impact on cell proliferation against NIH 3T3 fibroblast cell line. In addition, insulin was loaded into the glycopolymer particles due to both hydrophilic and hydrophobic interactions, with loading capacity and encapsulation efficiency of 9–11 and 60%–80% approximately, depending on the composition. Afterwards, this group [51] also synthesized a random amphiphilic glycopolymer of AAPBA with 2-lactobionamidoethyl methacrylate (LAMA). [51] The self-assembly of this copolymer was able to highly load insulin (loading capacity and encapsulation efficiency of 12% and 90%, respectively). In addition, they have proved that after nasal administration to diabetic rats, it can significantly decrease the glucose levels. 

**Figure 8:**
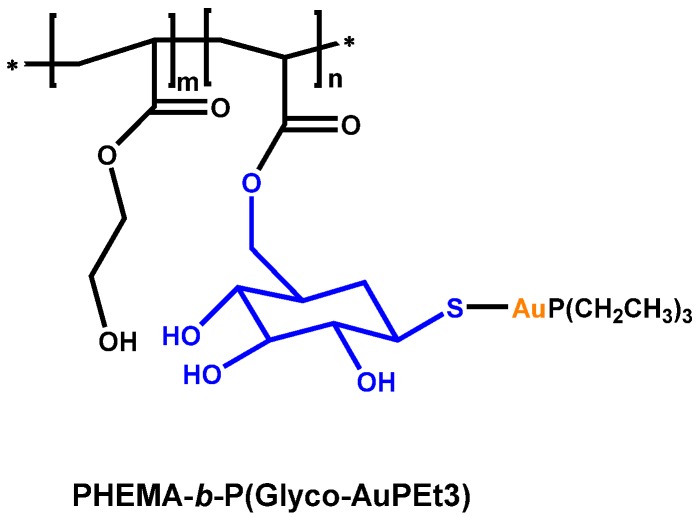
Block copolymer complex structure.

Stenzel group has synthesized homo and block copolymers with glycomonomer bearing protected thiosugar units. These systems can then be converted by post-modification into polymeric gold(I) complexes (Figure 8). The formed micelles were tested measuring the proliferation OVCAR-3 human ovarian carcinoma cells. [52]. The activity of the block glycopolymer complex is comparable to deacetylated auranofin or auranofin, which are potent antitumor drug. 

**Figure 9:**
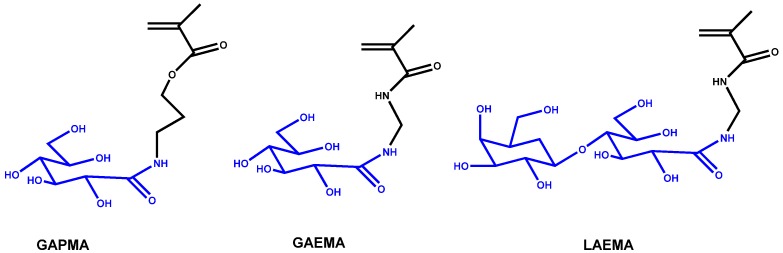
Different glycomonomer structures.

Likewise, Narain group also synthesized statistical glycopolymers of 3-gluconamidopropyl methacrylamide (GAPMA) (Figure 9) with 3-aminopropyl methacrylamide (APMA). These were subsequently modified with gold (I) phosphine to form the polymeric complexes [53]. The copolymer with lower molecular weight showed higher activity toward different cell lines (Hep G2, HEK 293T, MCF-7, and human dermal fibroblasts), especially toward MCF-7 cells. This polymeric complex presented higher accumulation and cytotoxicity in cancer cells under hypoxic conditions, which were used to mimic the therapy-resistant conditions in cancer diseases, in comparison to the normoxic conditions. Posterior, this group also synthesized statistical glycopolymers of 2-gluconamidoethyl methacrylamide (GAEMA), 2-lactobionamidoethyl methacrylamide (LAEMA) (see also Figure 9), and glycidyl methacrylamide (GMA) with different molecular weights [54]. These glycopolymers are decorated with dithiocarbamate to prepare conjugated glyconanoparticles with the anticancer drug, gold (I) triphenylphosphine, which were then used for targeted delivery to ASGP-R overexpressing HepG2 cells.

Fleming *et al.* [55] prepared a carbohydrate-antioxidant hybrid polymer using a different approach in order to deliver vitamin E, α-tocopherol, to porcine spermatozoa. The results demonstrated the interaction between the polymer and the galactose-binding protein presented on the mammalian spermatozoa, followed by the endocytosis and release of antioxidant. Consequently, an increase of fertilization rates by a diminishment of the degradation process is produced.

Series of diblock glycopolycations of 2-deoxy-2-methacrylamido glucopyranose (MAG), *N*-[3-(*N*,*N*-dimethylamino) propyl] methacrylamide (DMAPMA), and *N*-(2-aminoethyl) methacrylamide (AEMA) have been developed by aqueous RAFT [56]. These glycopolymers are all able to complex plasmid DNA into polyplex structures and to prevent colloidal aggregation of polyplexes in physiological salt conditions. Moreover, they are able to transfect in HeLa (human cervix adenocarcinoma) cells and HepG2 cells, being much higher in those with AEMA primary amine blocks and in HepG2 cells than in HeLa cells. Therefore, the glyco-block benefits the hepatocyte transfection and DNA delivery. 

A series of cyclodextrin-based mannose and fucose glycoconjugates, including glycoclusters, star glycopolymers and star diblock glycopolymers, have also been synthesized via combination of copper(I)-catalyzed Azide-Alkyne Cycloaddition (CuAAC) Huisgen coupling and ATRP. These glycoconjugates showed high affinity binding to the human transmembrane lectin DC-SIGN and act as inhibitors to prevent the binding of human immunodeficiency virus (HIV) envelope protein gp120 to DC-SIGN at nanomolar concentrations as demonstrated before. In addition, the star diblock glycopolymers showed high loading capacity of hydrophobic 1,4-dihydroxyanthraquinone and Saquinavir mesylate as anticancer and anti-HIV drugs, respectively. [57] 

## 5. Vaccines

Glycopolymers can also be good candidates to develop vaccines and to provide active acquired immunity against diseases [58,59]. A polymerizable version of the Tn-antigen glycan has been prepared and converted into well-defined glycopolymers by RAFT polymerization [60]. These glycopolymers were then conjugated to gold nanoparticles with narrow size distribution of diameter between 5 and 20 nm. Immunologically, these nanomaterials generated strong and long-lasting production of antibodies that are selective to the Tn-antigen glycan and cross-reactive toward mucin proteins displaying Tn. 

**Figure 10:**
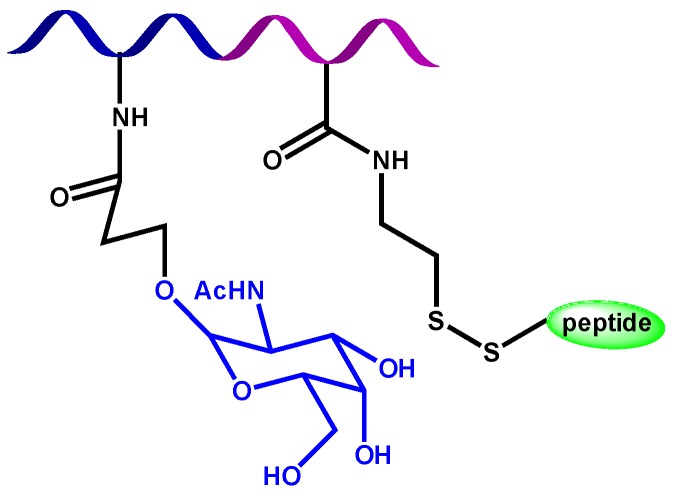
Glycopolymer vaccine structure.

Very recently, a block glycopolymer has been prepared by cyanoxyl-mediated free radical polymerization followed by conjugation with a tumor associated carbohydrate antigens (TACA) Tn antigen and a mouse T helper cells (Th cells) peptide epitope derived from polio virus to afford the vaccine construct (Figure 10) [61]. 

The glycopolymer vaccine stimulated an anti-Tn immune response with significant titers of IgG antibodies, which recognized Tn-expressing tumor cells. Although glycopolymeric platforms are still discreet, they offer great flexibilities to adjust antibody generator density, valence and the ratio of tumor associated carbohydrate antigens (TACA) against Th epitope. In addition, the immunogenicity of the glycopolymer backbone is not elevated, which probably will not compete significantly with the desired TACA for B cell activation.

## 6. Sensors

**Figure 11:**
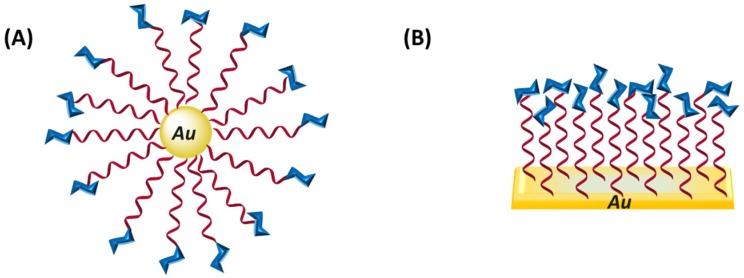
Schematic representation of glycopolymers attached to (**A**) gold particles and (**B**) flat gold surface, e.g., quartz crystal microbalance.

Due to the binding capacity of glycopolymers towards protein and bacteria, they have been extensively investigated as fundamental part of biosensors systems, mostly of them based on gold nanoparticles/surfaces and in their plasmonic properties. As commented above bacterial infections are today one of the people major concerns and glycopolymers can help in the solution as bacterial sensor. Gold nanoparticles with carbohydrates directly immobilized on their surface by Au-S bond formation showed very rapid responses to both lectins and lectin-expressing bacteria. In this sense, Richard *et al*. [62] have described a system capable of discriminating between different strains of *Escherichia coli* using this approach; that is, gold nanoparticles functionalized with poly(ethylene glycol) (PEG) with galactose and mannose-ended (Figure 11). The use of the PEG chains increases the saline stability retaining their biorecognition properties. The optical properties of nanoparticles change when bind to protein *Fim*H positive bacteria, enabling the identification of bacteria strain.

Other statistical glycopolymers having pendant thioglycosides have been synthetized by RAFT and then immobilized on gold nanoparticles as well as on quartz crystal microbalance (QCM). They showed strong and specific binding with their corresponding lectins, with estimated association constant (K_a_) values of around 10^7^ M^−1^ [63]. This value is reached with only 10% carbohydrate units, which gives an idea of the strong glycocluster effect (the interaction between lectin and free saccharide molecule is around 10^3^ M^−1^).

*p*-Acrylamidophenyl-α-D-mannopyranoside homopolymers and mannose-incorporating nanogel particles have been immobilized on nano-imprinted cyclo-olefin polymer films [64]. The strong and specific interactions between the mannose-incorporating polymers and Con A lectin were analyzed by monitoring the changes in the reflection intensity of the film. The nanogel particles showed a larger binding capacity compared with the homopolymers, and are able to detect low protein concentrations (with a detection limit of 6.0 ng/mL). Furthermore, the detection limit of the developed biosensor was lower than that of surface plasmon resonance sensor (1.43 mg/mL).

Very recently, amphiphilic block glycopolymers based on 2-O-methacryloyloxyethyl-(β-D-lactose) (Lac) and 4-pyridilmethyl methacrylate (PyMA) have been synthesized by RAFT process. Through the pyridine groups those can be chemo-adsorbed onto gold surface of QCM [65]. The constant value obtained for the specific binding with RCA_120_ is 6.26 × 10^6^ M^−1^. This approach was also employed to modify gold nanorods. A very small amount of lectin (100 pg/mL = 8.3 × 10^−13^ M) was detected by the aggregation of these glyco-gold nanorods, which gives idea of the potential of these systems [66].

Statistical glycopolymers of LAEMA and cationic 2-aminoethyl methacrylamide hydrochloride (AEMA) monomers obtained by RAFT polymerization were immobilized on a sensor surface for studies of bacterial adhesion by QCM with dissipation [67]. Galactose specific lectin RCA_120_ was used to test the QCM bacterial adhesion. Then, significantly higher amount of *Pseudomonas aeruginosa* PAO1 adhere on the glycopolymer surface with strong contact point stiffness as compared to *E. coli* K-12, since *P. aeruginosa* has galactose-specific binding while *E. coli* has mannose-specific one. Moreover, RAFT glycopolymers of acrylamide and glycomonomer bearing triazole-linked sialyloligosaccharides have been also immobilized on a gold-coated sensor of QCM. These glycopolymers strongly bind to both human and avian influenza A viruses [68].

**Figure 12:**
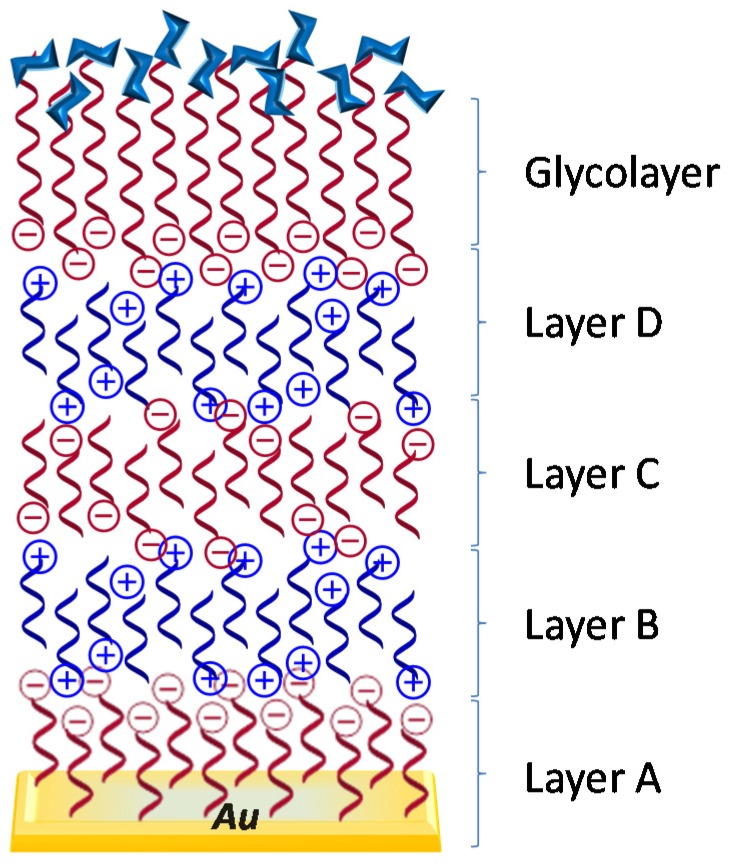
Schematic representation of layer-by-layer preparation of glycochips.

Layer-by-layer (LbL) is a widely used technique for thin film preparation. It consists of the alternate deposition of interacting species on a substrate with an intervening rinsing step following each deposition [69]. Usawa *et al.* [70] employed LbL adsorption methodology for the assembly of glycochips by using polyanionic glycopolymers (Figure 12). They prepared three glycochips carrying globobioside (Gb2), β-lactoside (β-Lac), or α-D-mannoside (α-Man) residues for the detection of Shiga toxins, Stx1 and Stx2, by surface plasmon resonance (SPR). Stx1 and Stx2 toxins show binding specificity for the Gb2 glycochip and a weak affinity for the β-Lac glycochip. Therefore, they give different SPR response allowing discriminating between the two toxins.

## 7. Imaging and Diagnostic 

### 7.1. Glycopolymers

The early detection of tumors or other illnesses are attracting ongoing attention. Nowadays, the development of systems with high cellular internalization efficiency and specificity, as well as low toxicity and good stability is of urgent need. Related to this, the conjugation of glycopolymers with contrast agent probes is a very promising alternative to achieve these purposes [71]. Wang *et al.* [72] have terpolymerized glucose- and lactose-containing methacrylate (GEMA and MAMA) monomers with radiopaque 2-(2’-iodobenzoyl)-ethyl methacrylate (2-IEMA) in presence of methyl methacrylate (MMA) as target imaging carrier materials for controlled drug release. These materials are thermally stable and a 0.50 mm thick terpolymer disk exhibits radiopacity similar to a 0.88 mm thick aluminum plate. Both glycopolymeric systems are radiopaque but the most of them is the one based on LAMA with a molar fraction of 0.5. Computed Tomography (CT) showed that aqueous solution of terpolymers with glycopolymer compositions of 0.5, especially lactose derivative, enhances visibility in the liver and kidney organs. CT values demonstrated that glycopolymer stays in kidney organs for at least one hour without an appreciable loss of contrast. 

Nevertheless, the conjugation with fluorescent probe is probably the most investigated strategy. Mannose-derivative of 2-hydroxyethyl methacrylate (MEMA) glycomonomer was copolymerized at different molar fractions with HEMA to analyze the ability to interact with Con A [73]. Besides, it was copolymerized with fluorescence 7-(*N-*(4-vinylbenzyl)amino)-4-trifluoromethylcoumarin to visualize the cellular internalization via endocytosis without apparent damage to the HeLa cells. The no toxicity of the glycopolymer was demonstrated on the basis of the maintenance of NADH dehydrogenase activity of cells after incubation for 24 hours.

Another fluorescence glycopolymer has been synthesized by simultaneous atom transfer radical polymerization (ATRP) and click chemistry using as fluorescent initiator N,N’-bis{2-[2-[(2-bromo-2-methylpropanoyl)oxy]ethoxy]ethyl}perylene-3,4,9,10-tetracarboxylic acid bisimide [74]. The glycopolymers with different length emit strong green fluorescence and exhibit good biocompatibility with 3T3 fibroblasts, macrophages, and KB cells. Therefore, they can also be used as cell labeling agents.

In addition, glycopolymers with near infrared fluorescence have been synthesized employing a combination of ring opening polymerization and RAFT polymerization, followed by post-functionalization with an aminocyanine molecule [75]. These materials after incubation with HepG2 cells for 72 h showed low toxicity even at concentration as high as 0.25 mg mL^−1^. They undergo enhanced and fast endocytosis due to the specific interaction of galactose glycopolymer with HepG2 cells.

### 7.2. Hybrid Materials

The groups of Zhao and Chen have synthesized by RAFT process copolymers of MAG and methacrylic acid (MAA). They used these glycopolymers as templates to prepare fluorescent glycopolymer-functionalized silver nanoclusters (with particle size of ~5 nm) through microwave irradiation [76]. The bioactivity of these systems has been evaluated using GLUT-1 over-expressing cancer cells K562, showing great toxicity over them. Furthermore, they have obtained porphyrin-glycopolymer conjugates based on this MAG glycomonomer. Glycoparticles (~200 nm) showed enhanced binding ability toward Con A, due to the stronger multivalent effect displayed by the sufficient numbers of glucose groups on the surface of the nanoparticles. They were also able to efficiently bind cancer cells K562 and kill them under light irradiation. Therefore, these systems can be used as photosensitizer for cancer imaging and also in photodynamic therapy [77].

In addition, star-shaped porphyrin-cored block copolymers based on gluconamidoethyl methacrylate (GAMA) have been also synthesized by RAFT using as macroinitiator poly(L-Lactide) with anchored porphyrin (SPPLA) [78]. These glycopolymers showed specific recognition with Con A. Furthermore, SPPLA-*b*-PGAMA_26_ block copolymer exhibited efficient singlet oxygen generation and high fluorescence quantum yields. Its cytotoxicity against COS-7 cells was very low and, when given a longer irradiation time, more BEL-7402 cancer cells died. Consequently, it can be useful for photodynamic therapy. 

Glycopolymers have also been combined with boron-dipyrromethene (BODIPY) for direct tumor cell imaging [79]. Statistical glycopolymer showing high water solubility based on galactose-derivative of 2-hydroxyethyl methacrylate (GaEMA) glycomonomer and BODIPY fluorescent dye monomer was developed. It presented good photo-stability and no cytotoxicity against HepG2 and NIH3T3 cells. The viability of cells was higher than the corresponding parent copolymer based on 2-hydroxyethyl methacrylate (HEMA) and BODIPY, indicating that galactose units decrease the toxicity. Moreover, its fluorescence quantum yield was 1.7 times higher than that of a BODIPY monomer because of the incorporation of galactose. Besides, this glycopolymer leads to efficient internalization into HepG2 and clear visualizations in cytoplasm, distinguishing between HepG2 and NIH3T3 cells.

Very recently, glycopolymers of 1-(methacrylamido) glucopyranose (1-MAG) fluorescently labeled with fluorescein isothiocyanate (FITC) have been synthesized by RAFT process. Additionally, gold nanoparticles with poly(*N*-hydroxyethyl acrylamide) attached onto the surface by thiol group have also been prepared [80]. These nanoparticles were subsequently reacted with glucosamine hydrochloride to generate glycosylated nanoparticles. None of these systems are hemolytic and do not induce morphological changes in the cells. Therefore, they can be used in diagnostics or therapies.

One step further described in literature is the preparation of magnetic nanoparticles covered with FITC-labeled glycopolymer yielded fluorescent [81,82]. In these publications the visualization of the nanoparticles by fluorescence imaging is studied, but the possibility of application in magnetic resonance imaging (MRI) is also opened. 

Magnetic nanoparticles represent one of the most important nanomaterials in the diagnosis and treatment of cancer. Nanosystems based on iron oxide nanoparticles are currently being applied as contrast agent in MRI [83]. The use of nanoparticles in cancer therapy is highly convenient because of so-called enhanced permeability and retention effect. The tumor vessels are altered in comparison with those in sane cells and the nanoparticles from the blood can be internalized better into tumor tissues, thus selectively accumulated. When this phenomenon is combined with other targeting strategies by incorporation of specific moieties the resulting nanosystems can exhibit enhanced diagnostic capacity for a more specific treatment and at the same time less aggressive to the human body as the required dose can be considerably reduced. The attachment of glycopolymers onto the iron oxide nanoparticles allows this selective localization of the nanoparticles concomitantly with an increase in the colloidal stability of the nanoparticles [84]. Several studies are found in literature concerning the functionalization of magnetic nanoparticles with glycopolymers for MRI applications. A series of diblock glycopolymers based on PEG and different carbohydrates, α-D-mannose, α-D-glucose and β-D-glucose, have been attached to iron oxide nanoparticle surfaces and the influence of the binding ability of the resulting nanosystems on the MRI signal was evaluated [85]. Remarkably, the α-D-mannose functionalized magnetic nanoparticles showed an improved cell uptake in a lung cancer cell line (A549) and also exhibited high r_2_ transverse relaxivity when measured in a 9.4 T MRI. More important, the binding of the nanoparticles to the lectin Con A produces a significant change in T_2_ relaxation, which is proportional to the lectin concentration, thus enhancing the diagnosis future perspectives. 

In another study, iron oxide nanoparticles have been functionalized with poly (vinylbenzyl-O-β-D-galactopyranosyl-D-gluconamide) that contain galactose units, thus able to be recognized by asialoglycoprotein receptors on hepatocytes [86]. *In vivo* experiments in rats show that after injection the signal intensity of liver largely dropped on T_2_-weighted MR image revealing that the nanoparticles are mainly accumulated in liver. Therefore, these nanoparticles can present a potential as contrast liver-targeting MRI contrast agent.

## 8. Future Developments and Conclusions

Up to here, promising applications of glycopolymers are presented; however, this type of materials can also be used in very different matters that could lead to a number of future applications. This is the case of glycopolymers used to optically observe the conformations of structurally well-defined polymers anchored to fluid lipid membranes [87]. Remarkable phase control on the crystallization of calcium carbonate by the stereochemistry of glycopolymers has been achieved. The selection of phase is based on the chelating character of the hydroxyl groups of the pyranoses (glucose or galactose) and their individual orientation, which allow to the control biomineralization processes [88]. Recently, ionotropic gelation has been reported on glycopolymers synthesized by copolymerization of 2-hydroxyethyl methacrylamide and acrylamide- and methacrylamide-type macromonomers obtained by modification of alginate-derived oligosaccharides [89]. These hydrogels formed under mild conditions have potential applications in cell encapsulation and *in vitro* 3D cell culture. Moreover, glycopolymers are highly hemocompatible and do not induce clot formation, red blood cell aggregation, and immune response, thereby these macromolecular structures could be very useful in many different *in vivo* applications [90]. In summary, these materials are promising systems for diverse biological, biomedical and other related activities. In this sense, the cost-effective and straightforward synthesis of adequate glycomonomers and glycopolymers is essential; therefore, chemistry as well as its combination with different areas, such as biology and biomedicine to understand their behavior, mathematics, and physics to model, such as behavior, analytical, and nanotechnology to implement their properties, and others, will allow enlarging the glycopolymeric material applications. 
